# Supplementary material for: MetaRibo-Seq measures translation in microbiomes
Source: Nat Commun. 2020 Jun 29;11:3268. doi: 10.1038/s41467-020-17081-z (PMC7324362; doi:10.1038/s41467-020-17081-z)
Supplement: Supplementary file 10 — Supplementary Data 7 [file 41467_2020_17081_MOESM10_ESM.zip › File2/Confidence_High_Taxonomy/378722_out.krona.html]

Javascript must be enabled to view this page.

members
magnitude
magnitudeUnassigned
count
unassigned
taxon
rank

378722\_out

26

26
superkingdom
2

1239
26
phylum

186801
26
class

186802
26
order

186803
family
24

genus
19
841

7
species

SRS014412\_contig\_number\_contig-100\_19.130178SRS014923\_contig\_number\_46848SRS015264\_contig\_number\_5095SRS015890\_contig\_number\_26058SRS016335\_contig\_number\_48419SRS018984\_contig\_number\_33755SRS148253\_contig\_number\_contig-100\_267.100090
166486

species
12
360807

SRS016954\_contig\_number\_28031SRS048870\_contig\_number\_contig-100\_948.117188SRS052027\_contig\_number\_13149SRS098881\_contig\_number\_14629SRS104693\_contig\_number\_4735SRS143417\_contig\_number\_13647SRS143780\_contig\_number\_30435SRS144135\_contig\_number\_20469SRS144714\_contig\_number\_contig-100\_494.172876SRS147271\_contig\_number\_41593SRS148784\_contig\_number\_28873SRS893259\_contig\_number\_contig-100\_202.54033

5
genus
572511

33038

SRS014459\_contig\_number\_contig-100\_1057.206915SRS015065\_contig\_number\_contig-100\_196.192874SRS016095\_contig\_number\_contig-100\_52.79555SRS022137\_contig\_number\_contig-100\_412.79674SRS058770\_contig\_number\_contig-100\_333.157593
5
species

2
family
31979

2
genus

SRS097920\_contig\_number\_14751
1
1485

59620

SRS024388\_contig\_number\_1162
1
species
